# Relationally competent attitudes and actions: a systematic review of general practice literature

**DOI:** 10.1080/02813432.2024.2417169

**Published:** 2024-10-20

**Authors:** Cæcilie Hansen, Ann Dorrit Guassora, Anne Beiter Arreskov, Annette Sofie Davidsen, Gritt Overbeck

**Affiliations:** a Centre for General Practice: The Section of General Practice Medicine and The Research Unit for General Practice, Department of Public Health, University of Copenhagen, Copenhagen, Denmark; b Innovation and Research Centre for Multimorbidity, Slagelse Hospital, Slagelse, Denmark

**Keywords:** Primary health care, general practitioners, physician-patient relations, attitude of health-personell, patient-centered care

## Abstract

**Objective:**

To explore core elements from Teachers’ Relational Competence in general practice literature regarding building relationships in consultations, specifying actions doctors take to create and maintain relationship quality with patients. This systematic literature review aims to map and propose a similar framework for the doctor-patient relationship.

**Background:**

The doctor-patient relationship, a well-researched yet complex field, often lacks clear descriptions. In recent definitions of patient-centred medicine, the responsibility of this relationship falls on the doctor, though how both relationship and responsibility is enacted needs clarification. Pedagogical literature on the student-teacher relationship provides a framework for relational competence, incorporating the needs and interactions between teacher and student and their alignment with institutional goals.

**Methods:**

A systematic review of two databases yielded 1256 hits. After screening, 15 studies were included and assessed. A qualitative synthesis was conducted through iterative and thematic deductive analysis.

**Results:**

Four relationally competent attitudes identified were: Attention to emotion, Devotion, Mentalization, and Time-oriented presence. Four relationally competent actions identitfied were: Being open, Attunement, Offering Support, and Using humor. Additionally, Trust and Continued connectedness were found as components of both attitudes and actions.

**Conclusion:**

An explanatory framework for professional relational competence for GPs includes concrete actions and specific attitudes before and during consultations. These consist of four key attitudes and four categories of actions with several subgroups of actions. Two additional components to the framework was found.

## KEY POINTS

- Patient-centredness is a concept widely known and used in healthcare, which doctors are expected to uphold, but some aspects of carrying out patient-centredness and relational work needs further research.
- Using inspiration from pedagogy, an explanatory framework can offer insights to how patient-centredness can be carried out in consultations
- The findings from our study can be used to further research into the doctor-patient relationship, as well as to strengthen education in communication in health care and consultations in general practice.
- This study found that an explanatory framework for professional relational competence for GP’s consists of concrete actions in the consultation as well as attitudes toward the consultation, both before and during the interaction

## IMPLICATION

The findings enhance existing conceptualizations of doctor-patient relationships in general practice and inform the development of these relationships. They provide a basis for further research and can be used to improve communication education in healthcare and consultations in general practice.

## Background

The scientific field of doctor-patient relationships is as long as it is wide; historically as long as the field of medicine itself, and conceptually wide in the variations of concepts which aim to investigate and describe the doctor-patient relationship. Some of the terms or concepts commonly used to describe and investigate this relationship are empathy and patient-centredness. These variations of concepts are, in themselves, very wide indeed. Empathy is essential to social relations but a human skill in all forms of relationships, and yet, it is used broadly in the literature on relationships in healthcare [1] as well as in psychology [2]. The use of empathy as a measure of doctors’ professionalism and communicative competence is questionable [3]) although attempts have been made to tailor it for clinical encounters [4]. Patient-centredness (PC), a concept widely known and used in healthcare, covers a broad set of skills and dimensions of care which doctors are expected to uphold [5–8]. The concept was launched by Enid Balint in 1969, based on the work of Michael Balint [9, 10]. He had introduced attention to psychodynamic aspects in the doctor-patient relationship and to seeing both the doctor and the patient as persons, with an emphasis on transference and countertransference [11, 12]. The concept is much alike to that of the person-centered approach, developed by Carl Rogers, also incorporated into general practice, with differences mainly pertaining to terminology rather than content [13–17]. Most of the newer research and definitions of PC focus on the therapeutic alliance [18, 19], sharing power and responsibility between doctor and patient [10], and on attaching importance to the patient as a person [5], which, too, finds resonance in the person-centered approach [16, 20, 21]. Langberg, Dyhr and Davidsen [5] suggest, based on a systematic review of definitions of PC, that its main elements could be narrowed down to (1) developing the doctor-patient relationship, (2) understanding the patient’s situation and, (3) managing coordination of care. The authors specify that the doctor has a responsibility for developing the relationship in PC; however, they also state that it deserves further attention how this responsibility can be carried out and developed.

Doctor-patient relations are often discussed in terms of continuity. Some suggest that the essence of primary health care is in the continuity involved in having a family doctor [22]. Relational continuity is known to reduce mortality, thus making it highly valued in general practice [23–25]. However, relational continuity in the strict sense of simply seeing the same doctor continually might not provide good doctor-patient relations in itself [26]. It is therefore unclear why relational continuity in the doctor-patient relationship works so well. To explore this ‘why’ might prove useful for cultivating relational continuity and thus enforcing the benefits of the doctor-patient relationship.

A developing area of study in the fields of education and teaching is dealing with professional and asymmetrical relationships through the lens of competency [27–29]. In pedagogy this is termed Teachers’ Relational Competence (TRC), and through this mode, researchers are able to concretize what the competence consists of. To be a teacher is to form and maintain quality in relationships with students in the class. This is considered an integral part of the job, and the single most reliable way to accomplish the goal of school as an institution; the specific goal in Danish secondary schools is formulated as ‘creating a conducive learning environment’ [29]. A main point is that relational competence is teachable, rather than an inherent one [28–30]. The definitions and core elements of Teachers’ Relational Competence are covered in a range of literature [28, 29, 31–36]. Please see overview of TRC in Table 1. The fundaments for TRC can be summed up into four separate components: attention to the teachers’ own prerequisites, attention to the students’ prerequisites, the interaction between teacher and student, and attention to the institutional goal [37]. For teachers, the relationship is not just important but essential, and the goal cannot be achieved without it. The relationship is also viewed as dependent on the teacher’s own preconditional involvement in the relationship, akin to what is implied in PC, yet not fully developed in general practice today [38].

**Table 1.** Literature on core elements in TRC informing the search strategy.

Core elements	Danish literature	Equivalent literature in English	Specific search word
Relational competence (the overarching concept)	Klinge 2016 [29] Juul and Jensen 2002 [34] Linder 2011 [32], 2018 [41] Fibæk Laursen 2004 [31]	Bringewatt et al. 2020 [30] Balint 1969 [9]	Relational competence, relational communication, interpersonal skills, socioemotional skills, Balint
Affect and emotion	Klinge 2016 [29] Linder 2011 [32], 2018 [41]	Aspelin et al. 2021 [42]	Transference, counter-transference, affect, emotional intelligence, compassion
Authenticity	Klinge 2016 [29] Juul og Jensen 2002 Fibæk Laursen 2004 [31],	*	Authenticity
Curiosity	Fibæk Laursen 2004 [31]	Aspelin et al. 2019 [28] Carl Rogers 1957 [14], 1995 [15]	Curiosity, Carl rogers, common therapeutic factors, person-centered psychotherapy,
Intersubjectivity	Klinge 2016 [29] Linder 2011 [32], 2018 [41] Juul & Jensen 2022	Fonagy, Allen & Bateman 2008 [43]	Interpersonal interaction, intersubjectivity
Moments of meeting	Linder 2018 [41]	Stern, 2004 [44]	Moments of meeting
Mentalization	Klinge 2016 [29]	Fonagy, Allen & Bateman, 2008 [43]	Mentalization
Psychological prerequisites or ‘stance’	Klinge 2016 [29] Fibæk Laursen 2004 [31]	Carl Rogers 1957 [14], 1995 [15]	therapeutic stance, therapeutic attitude, psychotherapeutic processes,

*No equivalent English literature informed on the concept of authenticity in TRC.

Departing from this notion about student-teacher relationships, that the quality in them is instrumental in order for teachers to do their job, might help narrow down what we mean, when we want to find out how quality in the doctor-patient relationship can be developed. To achieve this, there must to be a ‘language’ with which we can speak of it, to narrow it down and to make it visible for when consultations are analyzed.

### Aim

The aim of this systematic literature review is to explore which core elements from Teachers’ Relational Competence that appear in general practice literature concerned with building the relationship in the consultation, to specify which of the various things doctors do when talking to patients that creates and maintains quality in the relationship.

## Methods

To enhance transparency, we used ENTREQ reporting guidelines for systematic reviews [39].

### Search strategy

Our search strategy was developed with assistance from a specialist librarian and through discussion among the first, second, and last author. The search was conducted using the databases PubMed (Medline) and PsycInfo as sources. A comprehensive search strategy was formed using a PEO format [40] and by using an inclusive list of keywords, and synonyms related to core elements in TRC, see Table 1. The PEO parameters were population (general practitioners), exposure (core elements from TRC), and outcome (effect on the doctor-patient relationship and interaction in the consultation). Please see Supplementary Material for our exact search string. The core elements were those associated with the teachers’ prerequisites and the interaction between teacher and student from TRC theory [29, 36, 37]. Iterative test searches were performed for relevant and applicable keywords and controlled subject headings, as part of developing the search strategy, to ascertain whether a keyword would have relevant synonyms. Relevant search words for Balint and Carl Rogers was included in the search string as it was already known by the authors and partly comparable to TRC.

### Study selection

**Figure 1.**
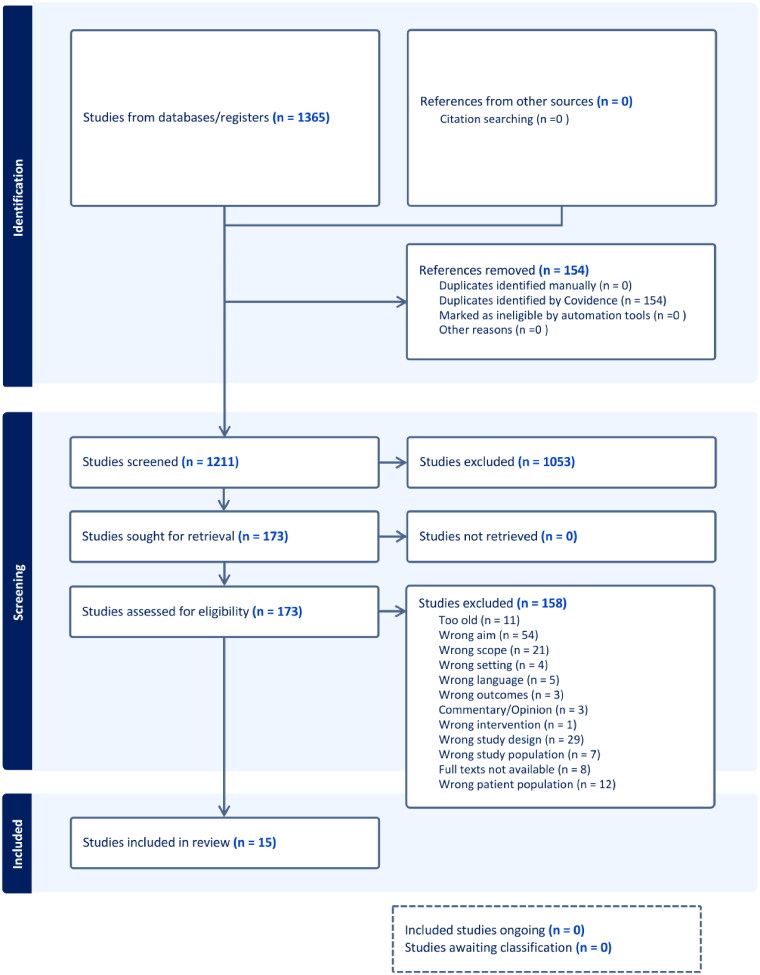
The PRISMA diagram.

The first and last author independently screened titles and abstracts of all retrieved articles using the Covidence software, after duplicates were removed in the software EndNote. When disagreement on inclusion occurred, authors met to discuss whether to include or exclude the article. This process was repeated with a screening of full texts. The first author then proceeded to analyze and code the included studies. The PRISMA diagram is shown in Figure 1.

### Inclusion and exclusion criteria

We included studies available in digital form, and studies published in paper form, if available. Studies were excluded if not written in English or Danish. We included studies on the doctor-patient relationship involving general practitioners but excluded studies with patients from a narrow population, such as only suicidal patients for example. Studies had to relate findings and results to the consultations performed in general practice. Studies were excluded if they did not relate to the consultation. We excluded studies published before 1980. Exclusion by study type was defined as other literature reviews, policy papers, protocols, and opinion pieces. Inclusion of study type was defined as studies informing on the consultation, whether qualitative or quantitative.

### Quality assessment

The articles were assessed with the reporting quality tools COREQ for qualitative studies [45] and STROBE for survey studies [46] by the first and last author. Range in qualitative articles assessed by COREQ was 18–29, maximum possible assessment being 32. Range in quantitative articles assessed by STROBE was 13–16, maximum possible assessment being 22. No studies were excluded based on the reporting quality assessment.

### Data extraction and synthesis of results

**Figure 2.**
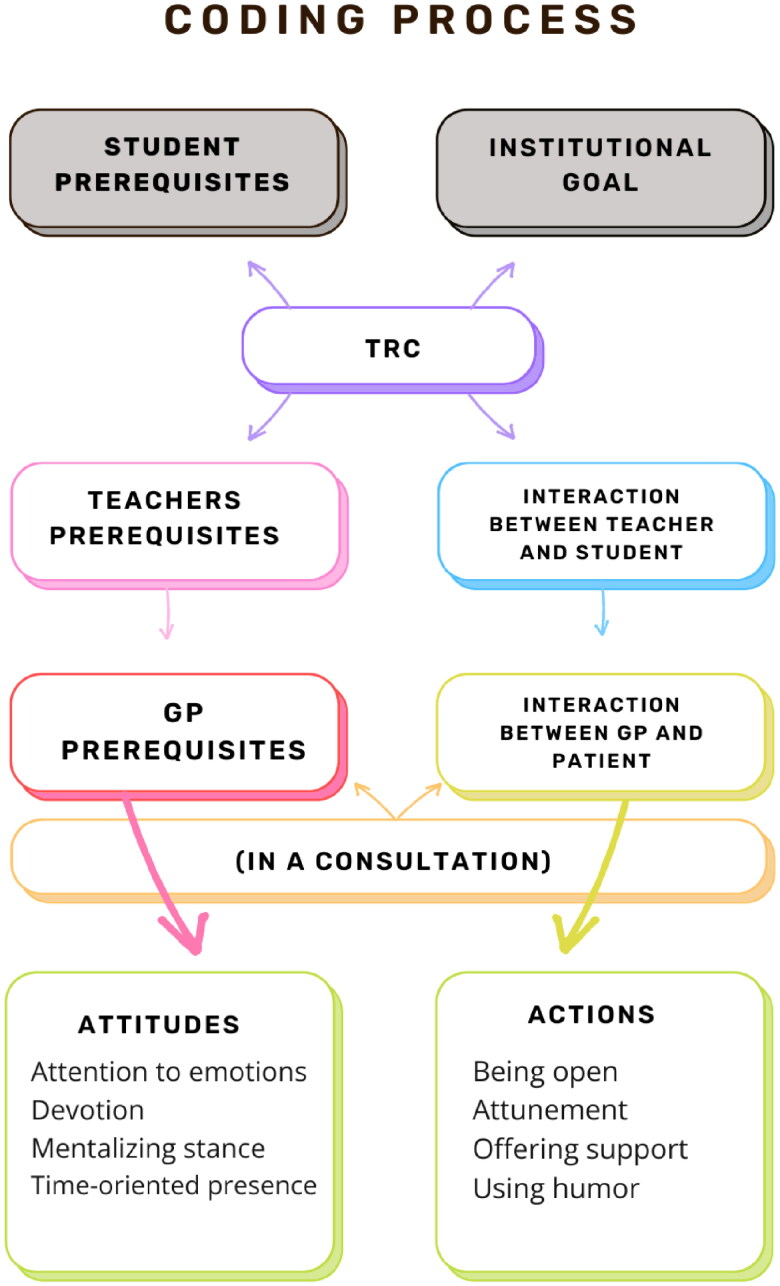
Chart showing the coding process with use of the unconstrained matrix.

Data extraction from the included studies was done through iterative full-text readings. The aim of this review was to explore the possibilities for a framework similar to that found in Teachers’ Relational Competence (TRC), which made exploratory deductive thematic analysis relevant. To maintain an exploratory approach, content analysis informed on the process of making a categorization matrix, which was used unconstrained in order to make room for relevant sub-categories under the generic categories [47]. The matrix consisted of two generic categories, *GP’s prerequisites* and *the interaction between GP and patient*, developed from TRC core elements in the components *Teachers’ prerequisites* and *interaction between teacher and student*. The other two components, *institutional goal* and *student prerequisites* were excluded from the search, as well as the coding matrix. To accommodate the different institutional setting, both *GP’s prerequisites* and *the interaction between GP and patient*, had to relate in some degree to general practice consultations, as it was an inclusion criteria (see Figure 2).

Extraction of data from the included articles was conducted through iterative full-text readings, to establish how the core elements taken from TRC were addressed. Included articles were read and analyzed qualitatively by the first, second and last author. For the analysis of the texts, thematic analysis [48] was used, and the unconstrained categorization matrix guided the search for relevant themes in the text. Thematic analysis helped in identifying latent themes in the text, which ended up fitting with the GP’s prerequisites in our matrix, and thematic analysis helped identifying themes on a semantic level which became fitting for *the interaction between GP and patient* in our matrix. The full-text readings were thus guided by the categorization matrix, but was unconstrained in order to maintain the exploratory approach stated in the aim.

The first author presented preliminary results which were subsequently discussed among the first, second, and last author. Iterative discussions of the interpretation of the findings were held among all authors, after the first, second, and last author presented suggestions for themes. The two generic categories from the matrix was renamed ‘attitudes’ (from GP’s prerequisites) and ‘actions’ (from interaction between GP and patient in a consultation) in light of the findings.

## Results

Our search yielded 1356 articles, as presented in Figure 1. 154 duplicates were removed and 1211 were screened by the first and last author. Full text screening was conducted of 173 studies by the same two authors, and in the end, 15 studies were included in the study. All included studies were in English and retrieved in digital form. Both qualitative and quantitative studies were included. The findings are divided into ‘Attitudes’ and ‘Actions’, which will reflect the *GP’s prerequisites* and *the interaction between GP and patient in a consultation* as seen in Figure 2. We found four attitudes, which are relationally competent attitudes for GP’s, describing a dual attention towards both the patient and the GP themselves, and how both parties influence the interaction, as well as the GPs’ responsibility for handling the relation in the moment and in future moments. We found four types of relationally competent actions for GP’s, pertaining to the dynamics of the conversation with patients, but also how to handle positions or opinions which diverge from patient disclosure on health related issues. An extra category in the findings, called ‘Benefits and Precursors’, deals with elements related to the findings that are products of or context for the first two categories. These elements are both results of building quality in the relationship as well as precursors for building more quality when having consultations with patients. We suggest that our results is a step towards developing a competence methodology for general practitioners, which we will call General Practitioners’ Relational Competence, GP-RC (Table 2).

**Table 2.** Included studies.

Author(s)	Publication year	Title	Country	Aim
Ben-Sira, Z.	1980	Affective and instrumental components in the physician-patient relationship: an additional dimension of interaction theory	Israel	The aim of the present study is to investigate the empirical support for the validity of a model, which shows the hypothesis that in the interaction between general practitioner and patient, the affective component of the physician’s behavior toward the patient (mode of behavior) will be a major factor in the latter’s assessment of the instrumental component of the physician’s behavior (content of behavior), i.e. the efficacy of the medical treatment.
Broholm-Jørgensen, M., Guassora, A. D., Reventlow, S., Dalton, S. O., & Tjørnhøj-Thomsen, T.	2017	Balancing trust and power: A qualitative study of GPs perceptions and strategies for retaining patients i preventitive health checks	Denmark	The aim for this study is to investigate how strategies of retaining patients are acted out by general practitioners (GPs) in the clinical encounter. Grimen’s (2009) analytical connection between trust and power is applied to explore how trust and power appear in preventive health checks from the GPs’ perspectives, and in what way trust and power affect and/or challenge strategies towards retaining patients without formal education.
Shaw, I.	2004	Doctors, ‘Dirty Work’ Patients, and ‘Revolving Doors’	UK	The aim of this study is to explore general practitioners’ perceptions of difficult patients and the consequences for patient management, using information from research into the phenomenon of ‘revolving-door’psychiatric patients.
Lundeby, T., Gulbrandsen,P., & Finset, A	2015	The Expanded Four Habits Model – A teachable conusltaion model for encounters with patients in emotional stress	Norway	The aim of this study is to develop a teachable consultation model for encounters with patients in emotional distress, by proposing a new expanded model to the original Four Habits model.
Davidsen, A.	2008	Experiences of carrying out talking therapy in general practice: A qualitative interview study	Denmark	The aim of the present study was to explore GPs’ experience of how they carried out talking therapy and to analyse if specific therapeutic approaches could be described.
Davidsen, A. & Fosgerau, C.	2014	General practitioners’ and psychiatrists’ responses to emotional disclosures in patients with depression	Denmark	The aim of this study is to investigate how GPs and psychiatrists, in consultations with patients suffering from depression, responded to patients’ emotional disclosures and whether or not they explored these disclosures.
Davidsen, A.	2009	How does the general practitioner understand the patient? A qualitative study about psychological interventions in general practice	Denmark	The present study aimed to explore GPs’ processes of understanding the patients with emotional problems. As there is no explicitly formulated theoretical framework for psychological interventions in general practice (Bower, 1998; Bower et al. 2001), an underlying idea of the study was also to find appropriate ways of conceptualizing the findings in a way that was relevant to general practice.
Mainous, A. G., III, Goodwin, M. A., & Stange, K. C.	2004	Patient-Physician Shared Experiences and Value Patients Place on Continuity of Care	USA	The purpose of this study is to examine the independent and interactive impact of longitudinal physician continuity and shared experiences of patients and physicians on patients’ desires for continuity of care by examining the relationship between the duration of relationship with the physician and a key event with physician (independent variables) with value of continuity (dependent variable) was analyzed using a 2-way analysis of variance while controlling for the confounding variables.
van Dulmen, S. & van den Brink-Muinen, A.	2004	Patients’ preferences and experiences in handling emotions: A study on communication sequences in primary care medical visits	Netherlands	The present study examined the communication sequences consisting of doctors’ responses to patients’ concerns in relation to the patient’s empathic preference and perception and the level of anxiety provoked by the medical visit.
Robinson, J. D., & Heritage, J.	2006	Physicians’ opening questions and patients’ satisfaction	USA	To determine the association between the format of physicians’ opening questions that solicit patients’ presenting concerns and patients’ post-visit evaluations of (i.e. satisfaction with) the affective-relational dimension of physicians’ communication.
Norfolk, T., Birdi, K., & Walsh, D.	2007	The role of empathy in establishing rapport in the consultation: A new model	UK	To highlight the role of empathy and communication skills in establishing rapport, we initially developed a model which seeks to draw the various motivational and skill elements identified in separate research papers into a comprehensive model of the journey towards shared understanding between doctor and patient. We then conducted an initial validation of the model *via* qualitative analysis involving general practitioners (GPs) and clinical psychologists.
Poon, V. H.	1997	Short techniques for busy family doctors	USA	To introduce two short counseling skills for busy family doctors: the BATHE technique and the DIG technique.
Uygur, J., Brown, J. B., & Herbert, C.	2019	Understanding compassion in family medicine: a qualitative study	UK	The purpose of this phenomenological study was to address some of the identified gaps in the extant literature by exploring family physicians’ perceptions, experiences, and ideas of compassion in the care of patients.
Davidsen, A. S., Guassora, A. D., & Reventlow, S.	2016	Understanding the body-mind in primary care	Denmark	The aim of this study is to describe the clinical situation in primary care and we will discuss different models that have been employed to understand patients when their symptoms traverse the gap between body and mind. We do this in an attempt to formulate concepts that could be useful for contributing to an understanding of patients’ experience of symptoms across the body–mind divide, and which could contribute to future integrative care in primary care and general medicine
Brown-Johnson, C., Schwartz, R., Maitra, A., Haverfield, M. C., Tierney, A., Shaw, J. G., Zulman, D. M.	2019	What is clinician presence? A qualitative interview study comparing physician and non-physicial insights about practices of human connection.	USA	The aim of this study is to define presence seeks to outline the important elements of clinician presence, and to specifically decouple it from patient-clinician communication, which is bi-directional. Clinician presence in our view can be enacted by physicians, with or without active patient reception. Although the term is commonly used, our research question centered on identifying a universal definition for clinician presence using qualitative data from interviews with primary care physicians and nonmedical professionals from diverse fields in which human connection is central.

## GPs’ relational competent attitudes

The following findings are attitudes the GPs can adopt when they enter the consultation. Some findings are more closely resembling actions, but they do however all involve attitudes towards patients on a macro level.

### Attention to emotion

Attention toward emotion in the consultation is described as a significant part of patient satisfaction, especially when the level of patients’ emotional concern is substantial [49, 50]. The attention to emotion can lead to higher satisfaction with medical procedures in the consultation [49] and to lower anxiety post-visit [50]. Attention to emotion is described to have effects such as *giving patients a feeling of reassurance* [49] and as *empathic qualities having been perceived in the GP* [50]. Attention to emotion can be performed by asking patients directly how they feel [51].

Attention to emotion has a counterpoint described as *medical irritation* [52], which is emotions experienced by doctors in certain situations and could prove harmful to the relation with patients if they are not handled properly. This could be similar to blaming the patient and projecting personal discomfort [53]. Medical irritation is proven to be unconscious, and the examples given by Shaw [52] describe relationships with patients that prove harmful to both the patient and the GP. Thus, this finding informs on a useful awareness for doctors to recognize their own feelings, by way of proving the consequences, should this awareness not be there.

### Devotion

Devotion is found to be a salient form of GP affective behavior in consultations [49]. Devotion is also a way of adding value to the relationship: one study describes the value of a continuous relationship when dealing with the patients’ problems as ‘being through a lot together’ [54]. Although the latter study does not directly mention devotion, it is an example of GPs being devoted to what the patient is going through continuously through time.

### Mentalization

The concept of mentalization can be useful when GPs want to apply a holistic view on patients’ minds and bodies, which can lead to ‘fruitful interactions’ [53]. Mentalization can be described as a ‘stance’, and deploying a mentalizing stance means that the GPs try to understand the patient’s mental state and motives as well as their own influence on the interaction. This can lead to more engagement in the consultation and more curiosity about and interest in the patient [55]. Another study mentioned curiosity, as an innate interest that helps GPs understand the patients and their life world [56], a description similar to that of a mentalizing stance.

### Time-oriented presence

Presence is a means to foster a connection with patients and this involves utilizing the time at hand to create this connection [57, 58]. This means that the GP must have attention to the available time and be present during this time. Presence is attention to moments where the GP understands something about the patients and their lifeworld during the consultation, which is conducive for connection to the patient [57]. Time-oriented presence is thus not only being in the room, but intentionally making the time spent with the patient count, and depending on how much time is available in the consultation, this utilization should happen relatively quickly.

## GPs’ relational competent actions

The following findings are categorized as concrete actions, although some of them are more or less concretely operationalized in the literature.

### Being open

Being open is described in several of our studies as different ways of interacting with the patient in the clinical encounter, and it is a behavior associated with quality in the doctor-patient relationship [50, 56, 58–61]. Being open is demonstrated through a validating language [58], accepting what the patient offers or the new possibilities arising in the consultation [56], using open-ended questions of the type ‘general inquiry’ [60], as well as investing in the beginning of the consultation [61], being quiet and listening while the patient is telling their story [59], and exercising an open body language [58, 59]. Thus, being open includes following the track of the patient, listening, and validating.

### Attunement

In several included studies, a phenomenon we term *attunement* appeared, referring to the recognition of and actively addressing the thoughts and feelings patients bring into the consultation. Attunement involves exploring the patient’s emotions and perspectives in the consultation [61], showing interest in them [49], and responding to emotional disclosures, thereby affirming the patient’s feelings [55]. Attunement means that the physician presents the opportunity to the patient to share their concerns with the physician. This expresses a stance of intention and wanting to hear the concern from the patient’s own mouth [60]. Attunement also entails that GPs should be aware that pre-formulated responses to patient concerns that are theoretically ‘adequate’ is not necessarily an adequate response in the actual consultation [50], meaning that a response can hardly be rehearsed, but has to be attuned to each patient from consultation to consultation. This is in line with findings of being sensitive to the specific patient and their resources [61]. The GP is attuning to patients’ thoughts, feelings, and expectations when they check, reflect, and mirror their own perceptions of the clues they pick up from the patient [56].

### Offering support

Offering support was described through a questioning technique which is designed to create a mutual understanding of the patient’s self-efficacy [51] and supporting the patient through empowerment. Offering support is also described as giving general encouragement to the patient [58]. To offer support and empowerment, GP’s must assess the patients’ strengths and resources [61]. Offering support is then the intentional verbal act of aiding patients to experience empowerment, achieved by assessing the patients’ strengths.

### Using humor

Humor was described as contributing to balancing respect for patients’ autonomy with respect for the GPs professional authority [62]. Humor can be a tool to let patients know the GP’s position or opinion in regard to health issues, such as smoking cessation, without simultaneously putting pressure on the patients to agree or change their position to match that of the GP. It was also described as a means to connect with patients [58].

## The benefits and precursors

During the synthesis of the studies in this literature review, we found two components that related to relational competence, but not something that was directly a part of the competency as such. It is not clear from the findings, whether these components are benefits *from* the sets of attitudes and actions or precursors *for* them.

### Trust

Trust was mentioned in several studies and described as an element that conditions the actions the GP takes in the consultation, but also as a result of the (beneficial) actions the GP takes. Trust is often referred to as a self-explanatory component of the GP-patient interaction [58], but some studies analyzed the dynamics of it. Trust is described as a means to retain patients and as something that creates a long-lasting relationship [62]. Trust is fostered through balancing the authority of the GP and respect for the patient’s autonomy, and this balance depends on the patient, the previous relationship, and the medical issue in question. Done successfully, the patient will want to come back, but if the right balance is not achieved, the patient will lose trust in the GP [62]. Trust is mentioned as a therapeutic component in the relationship, which allows the patient and doctor to engage in the consultation type ‘talking therapy’ [59]). Trust is also described as a result of the GP’s empathy and emotional awareness [57].

Trust seems to be both a component within the doctor-patient relationship and a result of it.

### Continuous connectedness

As stated above, trust is both a component and a result of quality in the doctor-patient relationship, and this suggests that quality in the relation is built over time. A lack of trust can damage the relationship, as trust has a spillover effect, benefiting future interactions or causing harm if absent. Continuous, demanding meetings for GPs may harm the relationship, potentially leading to a breakdown in continuity of care [51]. This highlights the importance of meaningful interactions, as sustained relationships with substantive content can foster quality. It seems that it is the continued connectedness through the trust in the doctor-patient relationship which sustains quality [54].

## Discussion

In the 15 included studies we identified core elements that constitute General Practitioners Relational Competence, GP-RC, which we divided into sets of *attitudes* and *actions*, as well as a set of precursors or benefits specific to the core elements we found. *Attitudes* were separated into four categories ranging from GPs’ emotional awareness towards patients, attention to own emotions, devotion, mentalization, and time-oriented presence. *Actions* that embed relational competence core elements were openness, attunement, offering support, and using humor. Additionally, we found that trust and continued connectedness are linked to the core elements, but it is unclear from the included studies if they predate the elements or are results of when the elements are observed.

GP-RC includes several dimensions which are not covered in PC, although PC is often used to describe the doctor-patient relationship. The attitudes *devotion* and *time-oriented presence*, and the actions *openness*, *attunement* and *humor* are not among the common aspects usually included in dimensions of PC [5]. This may reflect the knowledge from pedagogy of the professional’s primary responsibility for enabling the relation as a tool. It can inform this aspect of the GPs’ work. In PC, the dimension of ‘doctor as a person’ has been reduced gradually [5], but our results point to the potential of re-introducing the influence the doctor has in the relationship, which may even be healing [63], while also tethering it to the responsibility for the relationship.

Empathic qualities of the GP were included as a part of the attitude *attention to emotion*. While empathy is widely acknowledged in general practice as an important element in doctor-patient relations [3, 4], GP-RC seemed to include empathy but embraces several other attitudes and concrete actions, which play a role alongside the empathic qualities stated in the findings.

In the literature reviewed, continuity of care is described as a context for several of the core elements that appeared in the findings, but not as an attitude or action. We found that the value of the relationship is in the content or connection in the meeting itself, rather than in the continuity of meetings alone. Continuity in a doctor-patient relationship hinges on the quality within that relationship. This supports previous skepticism towards the idea that a long relationship provides quality in itself [26]. Our findings also support Rudebeck’s idea of relationship-based care in general practice [64] by giving details to his description of doctor-patient relationships as build and sustained throughout time, and that ‘good’ consultations will do good in the future.

In our findings, we operationalize a relationally competent action as involving attunement. It encompasses that the GPs check the understanding, reflect, and mirror the patient. Attunement is well known in therapeutic settings [65, 66]. In a report formulated by the Danish College of General Practitioners, a quote by Søren Kierkegaard is used to describe the importance of something similar to what we define as attunement [67]. The quote states the importance of ‘helping’ someone with attention to ‘finding them where they are’, meaning that in order to truly help someone, you have to assess what they are ready to receive help with. The quote is used in other articles relating to Danish general practice settings [68, 69], as well as in the curriculum for medical students, and the idea behind it is similar to the core of what we think attunement describes. The concept of ‘attunement’ helps operationalize how to ‘find patients where they are’. Other Danish frameworks use concepts bordering on attunement: the PRACTICAL framework [70] states the importance of adaption to each situation and person anew, as well as the concept of ‘receipts’ [71], which is a response model for GPs to attune to what the patient offers, as well as encouraging them actively to offer input on their experiences, by ‘giving a receipt to the patient’ that their concern has been heard.

Humor as an action proved to be the least described, mentioned in two studies but only with examples in one of them, yet operationalized as a concrete action to maintain the doctor’s relationship to his or her patient. Perspectives on humor in a medical setting, with its inherent complexities, are occasionally dealt with in The Lancet, demonstrating its’ presence in doctor-patient relationship discourse [72, 73]. Physicians using humor in medical encounters is perceived by patients to happen often [74], suggesting that it humor is intuitive, which only adds to the complexities. It is most often used in counselling to foster connection and bring warmth [75], suggesting that humor can be useful as emotional regulation. Types of humorous remarks can be roughly categorized as either benign or injurious, and benign humor is suggested as ‘safe’ in clinical settings [76]. This suggests that although humor is scarcely described in our findings, using humor has investigative potential and could prove useful for refining the understanding of GP’s relational competence.

### Strengths and Limitations in relation to the methodology of the review

The core strength of this review is the laborious methodology applied to the title and abstract screening following the development of a broad and varied search string. This systematic review is, as far as we are aware, the first to search for Teachers Relational Competence core elements in general practice literature. By applying a larger number of terms and concepts from TRC to establish which similar concepts exist in the general practice setting, we ensured a broad view on the literature. We also used two search columns to further broaden the search.

We did however limit our literature search to exclude grey literature. During the planning of the systematic search, it quickly proved too extensive to include grey literature with the breadth of our search string.

A limitation in the present study is the process of looking for similarity between pedagogical and medical concepts, by taking a departure in pedagogy. This will inevitably favor the pedagogical descriptions of the concepts which we tried to locate in general practice literature. This systematic review might then miss concepts in medicine similar to the core elements in TRC that are described using different terminology, like only being able to see where you shine a flashlight in the dark.

The absence of studies on ‘authenticity’ might be an example of this limitation. We included this keyword with the relevant synonyms and available controlled headings, but the concept was noticeably absent in the screening process. An explorative search to establish an existing concept for ‘authenticity’ could have proved useful.

It is, however, also worth noting that it is not the first time humanistic or behavioral science has been used to inform intervention in health settings or education of health care professionals [77–79]. This study fits with this tradition and should be interpreted as such.

### Strengths and limitations related to the included studies

The studies included in our study all take place in a primary care setting as a context, and the patient populations span over chronic care visits, acute care visits, patients with low socio-economic status, regular attenders with a history of psychiatric hospital admittance, and patients with depression or emotional problems. Some studies dealt entirely with the perspectives and experiences of GP’s and thus dealt with ‘patients’ as a presumably generic group. All studies inform on research pertaining to the consultation, and do not deal with other forms of doctor-patient interaction.

A strength is the variety of methodology in the included studies. This variety spans interview studies, observational studies, questionnaire studies, theoretical studies, and conversation analytical studies, thus supporting our search strategy that aimed for a broad view on the literature. The variety of methodologies provide volume, depth and theory in data.

The included studies were primarily from countries with similar healthcare systems, such as Norway, Denmark, The United Kingdom, and the Netherlands, which offer universal healthcare. Three studies were published in the United States of America, where the healthcare system is a mix of public and private healthcare and patients rely on medical insurance for access to care. This slight heterogeneity might be a limitation on the assumptions behind the relational possibilities in healthcare, when it comes to access. We do however see the flip side, the large homogeneity, to be a strength in this study, which counteracts the mixing of health care systems.

In our findings, one study proved significantly older than the rest, being published in 1980. This might prove this particular finding partly outdated, although the study in question scored 16 on the STROBE reporting quality assessment list. The content of the study is provided in several categories of our results, but most importantly *devotion and attention to emotion*, and as these contributions could not wholly be obtained elsewhere, we ultimately decided to incorporate it into our findings.

## Conclusion

This study found that an explanatory framework for professional relational competence for GP’s consists of concrete actions in the consultation as well as attitudes toward the consultation, both before and during the interaction. The elements consists of four attitudes, and four categories of actions, that gathers multiple actions in types. We found four attitudes, which are relationally competent attitudes for GP’s, describing a dual attention towards both the patient and the GP themselves, and how both parties influence the interaction, as well as the GPs’ responsibility for handling the relation in the moment and in future moments. We found four types of relationally competent actions for GP’s, pertaining to the dynamics of the conversation with patients, but also how to handle positions or opinions which diverge from patient disclosure on health related issues. We also found that surrounding the attitudes and actions, is trust and continued connectedness, which both are results of building quality in the relationship as well as precursors for building more quality when having consultations with patients.

## Implications

GP-RC adds to existing conceptualizations of doctor-patient relationships in general practice and to the operationalization of how to develop the doctor-patient relationship. The findings from our study can be used to further research into the doctor-patient relationship, as well as to strengthen education in communication in health care and consultations in general practice. The steps this study has taken towards mapping out what Relational Competence for general practitioners looks like, will be carried further into research by our research group to develop tools and methodologies for cultivating the competence for GPs.

Bits of the framework we propose is already represented throughout general practice literature, but it is dispersed throughout the field. We propose gathering it in a theory of General Practitioners’ Relational Competence, or GP-RC for short. An assembled theory will be useful for teaching and developing such a competence for general practitioners, as well as conducting research into the matter. We believe our results offer elements to a framework for building quality in relationships with patients.

## Supplementary Material

Supplemental Material
