# Supplementary material for: Relationally competent attitudes and actions: a systematic review of general practice literature
Source: Scand J Prim Health Care. 2024 Oct 20;43(1):181–93. doi: 10.1080/02813432.2024.2417169 (PMC11834779; doi:10.1080/02813432.2024.2417169)
Supplement: Supplemental Material [file IPRI_A_2417169_SM4996.docx]

# Supplementary material

Searches made at 10.11.23, GMT+2 14:19

**Supplementary table 1: Search string, PsycInfo**

| ( (DE "Family Physicians") OR (DE "Primary Health Care+") OR (DE "Family Practice") OR (DE “General practitioners”) OR (TX “GP*”) OR (TX “Family Physician”) OR (TX “Family Practice”) OR (TX “General Practitioner*”) OR (TX “General Practice”) OR (TX “Primary Health Care”) OR (TX “Primary Care Physician*”) ) AND ( (DE “Rogers (Carl)”) OR (DE “Authenticity”) OR (DE “Transference”) OR (DE “Countertransference”) OR (DE “Interpersonal Interaction”) OR (DE “Common Factors”) OR (DE “Mentalization”) OR (DE “Affect”) OR (DE "psychotherapeutic processes") OR (DE "person centered psychotherapy") TX ("relational competence" OR "relational communication" OR "curiosity" OR “Intersubjectivity” OR “Carl Rogers” OR “Balint group” OR “therapeutic attitude” OR “therapeutic stance” OR “authenticity” OR “transference” OR “countertransference” OR “counter transference” OR “Moments of meeting” OR “Moment of meeting” OR “Interpersonal competence” OR “Common therapeutic factor*” OR “Mentalization” OR “Mentalisation” OR “emotional disclosure” OR “emotional intelligence” OR “socioemotional skills”) ) [Show Less](about:blank) | **Expanders** - Apply equivalent subjects  **Search modes** - Boolean/Phrase | [**View Results**](about:blank) (850) |
| --- | --- | --- |
| Search: **#1 AND #3** Sort by: **Publication Date**  ("General Practice"[MeSH Terms] OR "General Practitioners"[MeSH Terms] OR "Primary Health Care"[MeSH Terms] OR "physicians, primary care"[MeSH Terms] OR "physicians, family"[MeSH Terms] OR "Family Practice"[MeSH Terms] OR "General Practice"[Text Word] OR "general practitioner*"[Text Word] OR "Primary Health Care"[Text Word] OR "primary care physician*"[Text Word] OR "Family Practice"[Text Word] OR "family physician*"[Text Word] OR "gp"[Text Word]) AND ((("relational communication"[Text Word] OR "relational competence"[Text Word] OR "curiosity"[Text Word] OR "intersubjectivity"[Text Word] OR "Carl Rogers"[Text Word] OR "Balint"[Text Word] OR "Balint group"[Text Word] OR "Therapeutic attitude"[Text Word] OR "Therapeutic stance"[Text Word] OR "authenticity"[Text Word] OR "Transference"[Text Word] OR "Countertransference"[Text Word] OR "Counter transference"[Text Word] OR "moments of meeting"[Text Word]) AND "moment of meeting"[Text Word]) OR "Interpersonal competence"[Text Word] OR "common therapeutic factor*"[Text Word] OR "mentalization"[Text Word] OR "person centered psychotherapy"[Text Word] OR "psychotherapeutic processes"[Text Word] OR "mentalization"[Text Word] OR "mentalisation"[Text Word] OR "emotional disclosure"[Text Word] OR "emotional intelligence"[Text Word] OR "Socioemotional skills"[Text Word] OR "Interpersonal interaction"[Text Word] OR "mentalization"[MeSH Terms] OR "person centered psychotherapy"[MeSH Terms] OR "psychotherapeutic processes"[MeSH Terms] OR "affect"[MeSH Terms]) | [515](https://pubmed.ncbi.nlm.nih.gov/?term=%231+AND+%233&sort=pubdate) |  |

**Supplementary table 2: Search string, Pubmed**
